# “In My Mind, It Was Just Temporary”: A Qualitative Study of the Impacts of Cancer on Men and Their Strategies to Cope

**DOI:** 10.1177/15579883231215153

**Published:** 2024-01-05

**Authors:** Corentin Montiel, Nathalie Bedrossian, André Myre, Asher Kramer, Alexia Piché, Meghan H. Mcdonough, Catherine M. Sabiston, Anika Petrella, Lise Gauvin, Isabelle Doré

**Affiliations:** 1 Université du Québec à Montréal, Montréal, Quebec, Canada; 2 Centre de recherche du Centre hospitalier de l’Université de Montréal, Montréal, Quebec, Canada; 3 Peer Researcher, Montréal, Quebec, Canada; 4 University of Calgary, Calgary, Alberta, Canada; 5 University of Toronto, Toronto, Ontario, Canada; 6 University College London Hospitals NHS Foundation Trust, London, UK

**Keywords:** oncology/cancer, coping, self-management, well-being, supportive care

## Abstract

Individuals who are diagnosed and treated for cancer use a variety of strategies to manage its impacts. However, there is currently a lack of research on men’s experience with managing cancer impacts, which is necessary to better support them throughout the cancer care continuum. This study explored the experience of men diagnosed with cancer, focusing on the impacts of the illness and its treatment and men’s strategies to cope. A qualitative descriptive design was used. Thirty-one men (*M_age_* = 52.7 [26–82] years) diagnosed with various cancer types were recruited to take part in individual telephone interviews (*n* = 14) or online focus groups (*n* = 17) addressing the impacts of cancer and strategies they used to cope with these impacts. Directed content analysis was performed, using Fitch’s (2008) supportive care framework to guide the analysis. Cancer impacts and strategies used to cope were classified into six categories: physical, psychological, interpersonal, informational, practical, and spiritual. Results indicate that the cancer experience is diverse and multifaceted rather than homogeneous. Medical and supportive care services could be more effectively personalized to meet the diversity of men’s needs by adopting a comprehensive and holistic approach to supportive care. Working in partnership with patients, it appears promising to recognize and identify men’s needs and match them to appropriate resources to provide truly supportive care.

Cancer is a leading cause of premature death worldwide (F. Bray et al., 2021). As populations grow and age, cancer incidence, prevalence, morbidity, and mortality are expected to increase (Sung et al., 2021). Despite the improvement in prevention and treatment protocols, the number of people surviving with cancer is steadily increasing. Lung cancer is currently the most frequently occurring cancer among men worldwide, followed by prostate cancer and colorectal cancer (Sung et al., 2021), while testicular cancer is the most common type of cancer in young men in industrialized nations (Giona, 2022). Although cancer survival rates have been increasing (Allemani et al., 2018), cancer remains a life-threatening disease, the diagnosis of which is often life-changing and leads to significant consequences in various life domains.

The impacts of cancer are diverse and can affect individuals in many ways. Cancer and its treatment can have significant physical consequences including pain, fatigue, and other unpleasant symptoms (Brearley et al., 2011). Alongside physical challenges, people living with cancer often experience distress (i.e., emotional or psychological difficulties), with estimates reaching 46% in a geographically diverse North American sample (Carlson et al., 2019). Distress is especially common in younger patients (Chambers et al., 2017; Dax et al., 2022; Rincones et al., 2021), unmarried men (Dax et al., 2022), and men with lower income (Chambers et al., 2017). Partners of individuals with cancer also experience distress (Chambers et al., 2011). Cancer-related cognitive impairment is another common issue, sometimes long-lasting, and may manifest as difficulties with attention, concentration, memory, learning, executive function, and processing speed (V. J. Bray et al., 2018; Lange et al., 2019). In part due to the important physical and psychological changes, men report significant interpersonal difficulties, including difficulties accepting and adapting to their new sexual and intimate reality (Collaço et al., 2018) and fear or worry about forming new intimate relationships (Dax et al., 2022). For young adults who are maturing socially, establishing identities, and seeking autonomy, the impacts of cancer on employment, social connections, and independence can significantly impact well-being and have long-lasting effects (Warner et al., 2016). Owing to challenges imposed by cancer treatments and their personal and financial costs, it is common for people diagnosed with cancer to face financial hardship and accrued debt (Altice et al., 2017). In addition, men experiencing severe symptoms may be unable to perform activities they value, such as physical activity, gardening, working, and social gatherings (Matheson et al., 2020). It is common for cancer to have a profound impact in terms of religious, spiritual, and existential concerns (Henoch & Danielson, 2009; Hvidt et al., 2019). Although religious and spiritual concerns are less prevalent in men than women (Hvidt et al., 2019), few studies address the prevalence and nature of these concerns in men.

Although few studies have focused on men, findings suggest that men use a variety of strategies to cope with the impacts of cancer (Cecil et al., 2010; Spendelow & Seidler, 2020; Wenger & Oliffe, 2014), with varying efficacy (Roesch et al., 2005). Wenger and Oliffe (2014) suggested that men with various types of cancer use multiple coping strategies, including building knowledge and physical strength, protecting others from distress, and restricting the expression of their emotions. A systematic review of qualitative studies in men with prostate cancer categorized strategies under four meta-themes; “avoidance, minimization, and withdrawal,” “directing cognition and attention,” “reframing masculinity and seeking support,” and “symptom/side-effects management” (Spendelow et al., 2018). Strategies to manage symptoms and side effects include medical and pharmacological interventions, lifestyle modification, social support, knowledge and information, navigation and coordination, and medical decision-making (van Dongen et al., 2020). One study in an outpatient cancer department offering information and support for self-management initiatives reported that exercise (84%) and diet (56%) were the most often used self-management strategies (Shneerson et al., 2015). Compared to women, men were less likely to use spiritual or religious self-management strategies or to use complementary or alternative medicine (Shneerson et al., 2015). Self-management strategies of men with prostate cancer included self-injecting, avoiding the exposure of treated areas to direct sunlight, good hygiene to reduce the risk of infection, maintaining a positive attitude, increasing rest periods, and getting help with chores from loved ones (Paterson et al., 2015). Along with partner support (Prashar et al., 2022), men appreciated the guidance from peers with cancer experience from whom they can receive advice, acquiring illness-related knowledge, anticipating obstacles, and experiencing reduced anxiety (King et al., 2015; Prashar et al., 2022; Wenger & Oliffe, 2014). Support from other men can be found in the context of support groups, but participation in such programs is low (Kim & Loscalzo, 2018), in part due to gender norms that influence men’s needs and preferences regarding communication and referral, as well as service goals, modalities, and delivery (Addis & Mahalik, 2003; Bender et al., 2012; Carter et al., 2014; Montiel et al., 2023; Neumann et al., 2010). For example, masculinity norms regarding emotional vulnerability may hinder men from participating in support groups, sharing their emotions, and tolerating negative and pessimistic discourses from other participants in such contexts (Montiel et al., 2023; Weber et al., 2000). Those who do participate report appreciating the activity and experiencing positive effects (Thaxton et al., 2005), as well as gaining knowledge, a sense of camaraderie, empowerment, and hope (Prashar et al., 2022). Finally, men appreciate the support provided by specialized nurses and physicians but may have limited access to these valued professionals (King et al., 2015; Wenger & Oliffe, 2014) due to administrative and clinical guidelines, or strained health care resources (Carter et al., 2014).

Studies addressing the impacts of cancer on men’s lives have mostly examined physical and psychological repercussions among older men with prostate cancer. Little is known about the impacts of cancer in other life domains, with men with other cancer types, and among younger men. Studies exploring strategies used by men with diverse cancer diagnoses are scarce; most studies have been conducted among women with breast cancer (Shneerson et al., 2015). Men are eager to manage their disease but remain “inadequately supported to do so” (Breau et al., 2003; Department of Health et al., 2010; Paterson et al., 2015). To support them, it is urgent to deepen our understanding of preferred and useful actions, strategies, and support men engage in.

This study examined the experience of men diagnosed with cancer, focusing on the impacts of the illness and its treatment and on men’s strategies to cope with the impacts. It is part of a larger project aimed at adapting the supportive care services offered to men by a hospital-based community organization.

## Methods

### Design

We used a descriptive qualitative design wherein data were collected through individual telephone interviews and online focus groups. The combination of individual interviews and focus groups was used for pragmatic reasons and to ensure data completeness (Lambert & Loiselle, 2008). Our study was grounded in a critical realism paradigm, which acknowledges the existence of an objective reality, but also recognizes that our interpretation of this reality is influenced by the social, historical, and cultural context, and that empirical observation alone may not be sufficient to fully grasp it. An advisory committee composed of two peer researchers (also referred to as patient partners), one health professional (kinesiologist), and the research coordinator CM (community psychology doctoral student) was first created to inform, consult, and collaborate during the various stages of the project. CM is a male in his late 20s who has experience in facilitating advisory committees, conducting qualitative data collection, and working with communities disproportionally affected by mental health difficulties. CM’s interest in the current research topic stems from his experience with marginalized and at-risk communities. He recognizes the importance of social and organizational factors and the promotion of macrosystemic change rather than putting the entire responsibility for behavior on the individuals. Peer researchers included two men (34 and 76 years old) who had been diagnosed with cancer in their life (kidney and prostate) and had received training in patient partnership involvement (Center of Excellence on Partnership with Patients and the Public, Montréal, Québec). The health professional was a former kinesiology intern at the partner organization, an active kinesiologist, and master’s student. In addition, the principal investigator (I.D.) and a research assistant (N.B.) were present during selected meetings. The study protocol was approved by the research ethics committee at the Center de recherche du Center hospitalier de l’Université de Montréal (CRCHUM certificate number 20.238). To ensure transparency, the consolidated criteria for reporting qualitative research (COREQ) checklist for qualitative studies according to EQUATOR guidelines is joint (Tong et al., 2007; see Supplementary File 1).

### Study Setting and Recruitment

Participants consisted of self-identified *men* aged 18 years and above who had received a cancer diagnosis during their lifetime, were living in the province of Québec, and spoke French or English. There was no exclusion criterion regarding sex assigned at birth. The convenience sample of men was recruited through various means including recruitment advertisements displayed on televisions inside the hospital; distributed through universities’ email lists; displayed on the hospital’s website and social media webpages; and shared on social media by local and provincial cancer associations, community organizations, and researchers. A paid algorithm-based Facebook advertisement was used to target men residing in the province who showed an interest in cancer by consulting cancer-related pages.

Men who contacted the research team were then informed by telephone of study details, research objectives, and implications. The first author took this opportunity to introduce himself and explain the research goals, his background, and his qualifications. Individual interviews were offered as an alternative to men indicating that they were not comfortable or interested in the group discussion format, men reporting nonavailability during focus group dates or men without internet access. When contacted, four men indicated a preference for individual interviews, two could not be grouped into focus groups due to availability, and three did not have internet access. After explaining the study and responding to questions, participants were directed toward a sociodemographic questionnaire on the Qualtrics platform (Qualtrics, Provo, UT). A written informed consent form was electronically signed before filling out the questionnaire. Based on the literature (Spendelow et al., 2018), recruitment was stopped when the sample size was thought to be sufficient to answer the research question in this study population (20–30 participants), and when new data collected stopped refining the main categories and subcategories.

### Data Collection

From March to May 2021, virtual focus groups were conducted in French by bilingual CM and one peer researcher using the Zoom platform (Zoom Video Communications, San Jose, CA). Focus group participants were stratified into three age categories (18–39, 40–54, and 55+ years) so that participants who were more likely to have similar diagnoses and experiences could engage in the discussion together. The interview guide (see Supplementary File 2) included open-ended questions about the impacts of cancer on their lives, their needs, strategies used to manage their experience, and the types of support they received and preferred. A financial compensation of $30 CAD was offered for the 2-hour discussion. After each focus group, the research coordinator and the peer researcher met for a short debriefing and note-taking task. Individual telephone interviews in French and English followed the same procedure and interview guide, though the interviewer was not accompanied by a peer researcher. Video and audio-recorded interviews were transcribed verbatim in the language used (all in French except one interview in English) and anonymized by an undergraduate student and checked for accuracy by CM before being uploaded to the qualitative analysis software MAXQDA (version 2020.4.2). Individuals received a compensation of $20 CAD for the 1-hour interview. All recordings were stored on the secured institutional server at the CRCHUM.

### Data Analysis

Owing to the complexity of the delimitation between, and the interactions among support, coping, and self-management (Audulv et al., 2016), all these different types of strategies were included and not differentiated. Fitch’s (2008) supportive care framework was used as a conceptual framework for the analysis and presentation of findings. The seven categories of cancer patient needs were slightly adapted to categorize the identified impacts and strategies, and presented separately to provide a comprehensive and detailed account of each. In this case, the *emotional* and *psychological* categories were merged due to conceptual similarity, and the *social* category was renamed *interpersonal* to better reflect the data content. Equal value was given to data collected by both methods, with individual participants representing the unit of analysis. Hsieh and Shannon’s (2005) directed content analysis with a manifest content approach was used following Assarroudi et al.’s (2018) 16-step method, including a preparation, an organization, and a reporting phase. In the organization phase, we first identified words, sentences, or paragraphs containing aspects related to each other through their content and context to extract meaning units. Meaning units were condensed before being merged into single codes in English. Clusters of codes were then categorized into main categories and subcategories. To improve credibility (Tracy, 2010; Whittemore et al., 2001), codes were reviewed independently by the research coordinator CM and a research assistant NB, and a consensus was reached when a divergence in coding arose. The coding tree structure was discussed with the principal investigator ID and research team members. A summary of the results was sent to all participants to give them the opportunity to provide feedback on the results.

## Findings

### Characteristics of Participants

Thirty-six men contacted the research team. Three men declined to participate after receiving information regarding the study due to a lack of interest. Two participants were excluded following data collection due to difficulties with oral communication following oral cancer which precluded participating in the interview (*n* = 1) and not meeting inclusion criteria (precancerous diagnosis; *n* = 1). A total of 31 cisgender men participated in the study (individual interviews *n* = 14; focus groups *n* = 17). The four focus groups of four to five men lasted an average of 127 [range: 123–131] minutes, while individual interviews lasted an average of 54 [range: 35–71] minutes. Sociodemographic and clinical characteristics are presented in Table 1. Men were aged on average 52.7 [26–82] years, *SD* = 14.72. Participants reported various cancer diagnoses including prostate (*n* = 6, 19.4% of men), testicular (*n* = 5, 16.1%), colorectal (*n* = 5, 16.1%), bones (*n* = 4, 12.9%), lymphatic system (*n* = 3, 9.7 %), kidney (*n* = 2, 6.5%), lung (*n* = 1, 3.2%), spine (*n* = 1, 3.2%), skin (*n* = 1, 3.2%), bile ducts (*n* = 1, 3.2%), throat (*n* = 1, 3.2%), intestinal (*n* = 1, 3.2%), peritoneum (*n* = 1, 3.2%), brain (*n* = 1, 3.2%), pancreas (*n* = 1, 3.2%), tonsils (*n* = 1, 3.2%), esophagus (*n* = 1, 3.2%), head and neck (*n* = 1, 3.2%), bladder (*n* = 1, 3.2%), and appendix (*n* = 1, 3.2%).

**Table 1.** Characteristics of the 31 Men Recruited to Participate in Focus Groups or Interviews About Their Experience of Cancer (Québec, Canada, 2021)

Characteristic	Individual interviews (*n* = 14)	Focus groups (*n* = 17)	All (*n* = 31)
	*n* (%)	*n* (%)	*n* (%)
Age group			
18–39	4 (28.6)	5 (29.4)	9 (29.0)
40–54	3 (21.4)	4 (23.5)	7 (22.6)
55+	7 (50.0)	8 (47.1)	15 (48.4)
Racial/ethnic group^a^			
White	12 (85.7)	17 (100.0)	29 (93.5)
Asian	1 (7.1)	0 (0.0)	1 (3.2)
Arab	1 (7.1)	0 (0.0)	1 (3.2)
Country of birth			
Canada	12 (85.7)	15 (88.2)	27 (87.1)
France	1 (7.1)	2 (11.8)	3 (9.7)
Philippines	1 (7.1)	0 (0.0)	1 (3.2)
Relationship			
In a relationship/married	10 (71.4)	12 (70.1)	22 (71.0)
Single	2 (14.3)	2 (11.8)	4 (12.9)
Widow/separated	2 (14.3)	2 (11.8)	4 (12.9)
Place of residence			
Montreal	8 (57.1)	7 (41.1)	15 (48.4)
Quebec City	5 (35.7)	2 (11.8)	7 (22.6)
Other	1 (7.1)	8 (47.1)	9 (29.0)
Occupation			
Full-time employment	5 (35.7)	9 (52.9)	14 (45.2)
Part-time employment	0 (0.0)	1 (5.9)	1 (3.2)
Retired	5 (35.7)	4 (2.4)	9 (29.0)
Unable to work/sick leave	4 (28.6)	3 (17.6)	7 (22.5)
Education (highest diploma)			
Did not finish high school	2 (14.3)	0 (0.0)	2 (6.5)
High school/trade school	5 (35.7)	0 (0.0)	5 (16.1)
Junior college (CEGEP)^b^	2 (14.3)	7 (41.2)	9 (29.0)
University	5 (35.7)	10 (58.8)	15 (48.4)
Cancer treatment phase			
In treatment	4 (28.6)	8 (47.1)	12 (38.7)
Post-treatment	9 (64.3)	9 (52.9)	18 (58.1)
No treatment available/terminal Dx	1 (7.1)	0 (0.0)	1 (3.2)
Cancer diagnosis			
Prostate	3 (21.4)	3 (17.6)	6 (19.4)
Testicular	2 (14.3)	3 (17.6)	5 (16.1)
Other	9 (64.3)	11 (64.7)	20 (64.5)

a Categories based on Statistics Canada and Census Canada.

b Collège d’enseignement general et professionnel (General and professional teaching college).

The impacts of cancer and strategies discussed by participants were categorized as physical, psychological, interpersonal, informational, practical, or spiritual. The definitions of these categories were then developed and adjusted according to the data. Within each domain, subcategories composed of clusters of codes are discussed with quotations to illustrate the meaning of the findings (see Tables 2 and 3). Quotations were all translated from French to English (except for those from one English-speaking participant). Quotations are identified by participant number and data collection type (FG = focus group, I = interviews), age, and treatment stage.

**Table 2.** Impacts in Each Domain Reported by 31 Men Recruited to Participate in Focus Groups or Interviews About Their Experience of Cancer (Québec, Canada, 2021)

Domain	Impact	Examples	Quotes
Physical	Body	Functioning, pain, fatigue, strength, weight	*You know when you get ID’d over at the convenience store, you show your card and the person says, “That’s not you on the license” because you don’t look like yourself anymore.—Participant FG03-01, 18-39 age group, testicular cancer.*
Psychological	Affect	Anxiety, fear, anger, relief	*And in a way I’m sad because I have this disease. I thought I would see . . . I thought I would see my grandchildren grow up, my daughters get married, you know.—Participant I11, 40–54 age group, colorectal cancer.*
	Cognitive processes	Irrational thoughts, loss of memory, self-reflection	*When you’re diagnosed with cancer, you immediately think, “When am I going to die?”—Participant I03, 55+ age group, bones, kidney cancers.*
	Attitudes	Becoming more authentic, increased creativity	*Whereas before, maybe I was trying too hard to please the people around me. I didn’t feel like I was 100% myself. So that’s changed in that respect, the experience that I had.—Participant I08, 40–54 age group, testicular cancer.*
	Sense of purpose	Appreciation of life, reconsideration of priorities	*And I got myself a glass of water and I drank it. And for the first time in my life, I realized, it’s silly, drinking a glass of water, but tomorrow I could lose that . . . how should I put it . . . that pleasure of having a glass of water. And I was drinking the glass of water facing my window above the sink and I looked outside. And I say it’s true that life is hanging by a thread, and all these little things, the pleasure whether it’s having a glass of water, whether it’s when I opened the door my dogs were waiting for me, whether it’s . . . All the little silly things we do in life that we don’t realize. I learned that they were little pleasures.—Participant I07, 40–54 age group, kidney cancer.*
Interpersonal	Relationships	Limitations on relationships, strengthening of relationships	Participant*—Already when you have cancer, you feel apart from others. For others, empathy is nice, it’s nice, but if they show me even more empathy, I feel even more isolated.* Interviewer*—In what sense?* Participant*—You know, it’s as if the world was spinning and I wasn’t part of it. I was just a witness. I don’t want to be a witness; I want to participate. —Participant I07, 40–54 age group, kidney cancer.*
	Social interactions	Lack of discussion topics, others being tired of cancer talk	*The discussions are difficult. I make them [friends] talk about their day, their work, but I don’t have . . . I can’t always talk about my illness, you know. The discussions . . . it’s different.—Participant FG01-03, 55+ age group, lung, spine, brain cancers.*
	Perceptions of others	Perceptions of being sick, fragile, brave	*So, when I got back on my two feet, got stronger, my hair grew back, it seemed like people kept the look of, “Ah that’s the one who got sick, that’s the one who got cancer, that’s the one . . .” —Participant I08, 40–54 age group, testicular cancer.*
Informational	Patient knowledge	Knowledge of health, becoming an expert of their illness	*And one of the skills that comes with it is navigating the healthcare system. You become an expert in the healthcare system [laughs].—Participant FG04-03, 40–54 age group, colorectal, peritoneum cancers.*
	Skills and abilities	Newfound skills and abilities	*Even if I don’t think that the disease makes you better, it allows you to develop certain skills.—Participant FG04-04, 40–54 age group, tonsils cancer.*
Practical	Activities	Difficulties engaging in physical activity and leisure activities	*But I can’t . . . I’ve tried to draw a little again, but I can’t hold my pencil without shaking. So, it’s not much fun when you’re used to drawing to not be able to draw and try to draw. I’ve moved on to other things. Even reading, honestly, it’s not always easy.—Participant FG04-03, 40–54 age group, colorectal, peritoneum cancers.*
	Occupation	Missing class, interruption of work	*Yes, that’s right, it was physical work. When I did too much, sometimes blood came out. It put a bit of a stop to the day. —Participant I11, 40–54 age group, colorectal cancer.*
	Financial situation	Drop in income, increased spending	*I think the financial side as well, in my case, well anyway I won’t go into details, but I had started a career as a self-employed worker so when it happened, I don’t want to complain more than anyone else or anything, but you know when you’re self-employed obviously you don’t have insurance, you don’t have a safety net. So that was a pretty big blow.—Participant FG04-01, 40–54 age group, colorectal cancer.*
	Chores and daily tasks	Impossibility of driving, difficulties doing household chores	*Well, you’re on morphine, you’re not there. As they say . . . it’s not the time to go . . . let’s say . . . just cooking my supper, I could burn it.”—Participant I11, 40–54 age group, colorectal cancer.*
Spiritual	Religious beliefs	Confirmation of atheism, newfound beliefs in God	*It actually made me realize my atheism [. . .]. Because I more or less almost died in fact, and I realized that I didn’t believe in God.—Participant I15, 18–39 age group, bones cancer.*

*Note.* FG = focus group.

**Table 3:** Strategies Reported in Each Domain by 31 Men Recruited to Participate in Focus Groups or Interviews About Their Experience of Cancer (Québec, Canada, 2021)

Domain	Strategy	Examples	Quotes
Physical	Health care and supportive care	Receiving medical treatment, nutritional counseling, physiotherapy	*Since I had lost a bit of time, I talked to her [doctor] about light therapy if it existed. Not if it existed, I knew it existed. She laughed and said, “I have a colleague who works on that, but it’s at [hospital 1],” the [hospital 2] was not yet open. So, they transferred me there. And he offered me treatment.—Participant I07, 40–54 age group, kidney cancer.*
	Lifestyle behavior changes	Adjusting lifestyles through supplements, oxygenation exercises, reducing alcohol consumption	*But I’ve completely changed my diet, I do a fast every two weeks, the Wim Hof method for daily breathing, by the book.—Participant I04, 55+ age group, prostate cancer.*
Psychological	Psychological support	Receiving psychological interventions, encouragement, comfort	*But it feels good to talk about it. There are things I tell her [psychologist] that I wouldn’t tell anyone. Sometimes I would say to her, “Ah, I’m sick of feeling a little bit sick, not just because of my cancer, but because of everything.” That I can say to her, but I wouldn’t say that to my kids.—Participant I10, 55+ age group, skin cancer.*
	Psychological processes	Positive outlook and mindset, enjoying life, denial, avoidance	*Pretty much nothing [impacts of cancer], because I didn’t want to. In my mind, it was just temporary. It’s a diagnosis, but it wasn’t going to attack me, it wasn’t going to change anything. It [my life] wasn’t going to come to an end, no way.—Participant I05, 55+ age group, prostate cancer.*
	Self-regulation behaviors	Relaxing, meditation, crying, looking for solutions	*You know, a little kick to say, well, okay, I’ll go to meditation. Yes, it’s true that a lot of women go there, but I met another guy who was there and listen, meditation did me good.—Participant FG01-03, 55+ age group, lung, spine, brain cancers.*
Interpersonal	Social affiliation	Being in the presence of others, ending relationships, forgiveness	*And I made the choice to put an end to that relationship for the sake of a better quality of life, and to put aside friendships that weren’t bringing me pleasant, positive things.—Participant I13, 18–39 age group, testicular cancer.*
	Managing others	Managing others’ emotions, putting their limits, helping others	*But there are lots of little things you can do for the people around you, for yourself and other things [. . .]. One of the things I do that makes me feel good is to try to make others feel good too.—Participant FG04-03, 40–54 age group, colorectal, peritoneum cancers.*
Informational	Managing information	Asking health care providers questions, avoiding online information	*And I’ve never asked my doctor about the percentages that it [cancer] comes back, or the percentages of recovery, because I don’t want to know.—Participant FG03-03, 18–34 age group, testicular cancer.*
	Learning	Learning to manage their condition, studying health-related topics	*So, I took MOOCs (Massive Open Online Courses) in medicine with the University of Paris VII or Paris IX, I think, which offered courses in both veterinary medicine and human medicine. So, at the same time, I was learning about different techniques, different things that affected me directly. So, on the MOOC side, I did a couple of courses. Not necessarily always successfully, but that’s not the point [laughs]. But in any case, it also gave me a better understanding afterwards, I did two or three.*
	Sharing	Sharing news with loved ones, sharing information with others cancer patients, sharing advice with male friends	*My cousin told me he had his biopsy, I told him “Don’t forget to tell me about it when you get your results, we’ll talk about the possibilities, see how the specialists see it, how I see it. We will talk.” I don’t start right away to get them thinking about what I’m talking about, diet, exercise, etc., because I know they’re not ready to make a big leap without knowing what’s going to happen.—Participant I04, 55+ age group, prostate cancer.*
Practical	Engagement in activities	Engaging in sports, leisure activities and hobbies, volunteering, house renovations	*And I really like making Legos (LEGO bricks). You know, very complex Legos. And my wife spoiled me a lot, every now and then she would give me a super complex Lego to make, and then I would spend hours and hours making Legos. During that time, I didn’t think about what had or what I was going to have or this or that, I just concentrated on making my Lego. And that was my way out.—Participant FG02-02, 55+ age group, prostate cancer.*
	Occupational adjustments	Switching to online classes, taking time off work, work adjustment	*When I left that company, I said, “When I come back, I’d like to be able to do two or three days a week” and the bosses told me “Certainly [participant’s first name], we don’t want to lose you.”—Participant I10, 55+ age group, skin cancer.*
	Financial support	Using governmental aids, savings, work benefits	*Fortunately, I had insurance. I can imagine people who don’t have insurance. Of course, I suppose there are government programs like unemployment insurance and the like. But it’s a good thing I had insurance at work.—Participant FG02-03, 55+ age group, lymphatic system cancer.*
	Help with tasks and chores	Friends bringing groceries, parents making dinner	*So, my friends all started telling me, “OK, we’ll go do your woodwork, we’ll take care of the animals, we’ll do this, we’ll do that, we’ll help your wife,” and they would bring recipes and food to help my wife.—Participant FG04-02, 40–54 age group, lymphatic system cancer.*
Spiritual	Religious involvement	Praying, receiving help from religious figures	*But I remember when I was diagnosed, I went to the church in the city where I live and I thought, ‘My God, someone has to help me.’ I still don’t practice. But in the back of my mind I think, maybe there is something up there.—Participant I03, 55+ age group, bone, and kidney cancer.*

*Note.* FG = focus group.

### Impacts of Cancer

Impacts of cancer were defined as the consequences of the cancer diagnosis and changes that have occurred since or are attributed to the cancer experience. Table 2 provides impacts identified in each domain, along with illustrative examples and quotes from participants.

#### Physical Impacts

Physical impacts included impacts on men’s bodies, physical comfort, freedom from pain, optimal nutrition, and ability to carry out usual bodily functions. Impacts on men’s bodies and their functioning were numerous and discussed extensively, and often in relation to the treatments received. For example, senses like vision, hearing, and taste were negatively impacted by treatments such as chemotherapy. Men indicated difficulties with executing movements and fine motor skills. Impacts on functioning specific to some diagnoses were reported, such as erectile dysfunction for those with testicular or prostate cancer. Men with prostate cancer could experience long-term incontinence and loss of libido, while some suggested a lack of physical sexual desire and performance issues. Other functional impacts included weight and urinary functions. Lack of strength was regularly discussed. Men mentioned the acute impacts of treatment on blood circulation, burns, nausea, night sweats, spatial disorientation, bleeding, hair loss, and others. Men also included more general and sometimes long-term physical impacts such as discomfort, scars, and an overall feeling of not being physically well.

#### Psychological Impacts

Psychological impacts were defined as cognitive, psychological, or affective changes and processes resulting from the experience of cancer. Men discussed primarily negative impacts on feelings, emotions, and moods (e.g., shock, anger, fear, hopelessness, worry, sadness, and uncertainty) related to several elements of the cancer experience (e.g., waiting time, bureaucracy, information on survival rates, current symptoms, and the possibility of long-term impacts). A majority of men reported generally “feeling down” and not feeling well emotionally, with some discussing the loss of pleasure, desire, and motivation to engage in activities. Most men also talked about some elements of positive affect, such as gratitude for good prognoses and relief from obtaining a diagnosis after a prolonged period of unexplained symptoms. Although less prominent, a few men noticed changes in their personality and attitudes since their diagnoses, such as being more authentic, less afraid of ridicule, more assertive, more patient, and more open to other ideas and values. Periods of intense motivation and inspiration were discussed but so were the negative impacts of their experience on self-esteem and self-confidence.

The cancer experience impacted men’s cognitive processes in terms of thoughts, reflections, and understanding. Most notably, a few men indicated questioning why they got cancer (e.g., smoking, working too much), while others found the origins of their cancer incomprehensible. Many men experienced difficult and challenging thoughts during their experience, including dark and irrational thoughts. These thoughts sometimes remained for a prolonged period. For example, hypervigilance about whether symptoms they experience are related to worsening or recurrence of cancer were difficult to eradicate, even after recovery. Cancer led men to reflect on life in general, including appreciation of life, its beauty, and its pleasures and perspectives on what is important in their lives (e.g., family rather than work).

#### Interpersonal Impacts

Interpersonal impacts included impacts on men’s interpersonal relationships, social networks, and community acceptance. Men reported several restrictions to their relationships, such as friends and loved ones avoiding being with them, talking to them, or limiting their interactions. Some men reported feeling excluded and isolated by others, even in their presence. The experience of cancer also weakened certain relationships, causing distance between some men and their friends, family members, and/or spouse, and led to the end of some relationships. They reported detecting discomfort in others, with people quickly changing the topic of discussion when talking about cancer, being tired of hearing about cancer, and being afraid of saying the wrong things. A few men discussed others constantly asking them about their cancer, saying the wrong things (e.g., “you are strong, you can fight this”), treating them differently, and being overly empathetic.

Alternatively, the cancer experience appeared to strengthen some relationships: many men discussed how they became closer to some people, particularly loved ones. Some men reported better interactions with others since their diagnosis and a better capacity to develop strong bonds with people. Men were often confronted with others’ incorrect, unwelcome, and often persistent assumptions, particularly regarding the difficulty of recovering from cancer and seeing them as sick, fragile, or going to die. However, some men experienced more positive reactions from others, such as admiration and being perceived as brave or positive throughout their experience.

#### Informational Impacts

Informational impacts were defined as the resulting consequences of going through the cancer care continuum on men’s knowledge, information, and skills. Some men report learning from their experience. The resulting knowledge gained of the health care system and medical health was discussed positively as it could potentially be mobilized in other situations (see strategies). Because of their cancer, men found themselves in a privileged place to learn. For some men, long treatments or recurring diagnoses meant they had to undergo aspects of the cancer care continuum multiple times. Only a few men briefly expressed that through this experience, they became experts in their illnesses, and their journey with cancer allowed them to develop new skills and abilities, though they did not expand on those.

#### Practical Impacts

Practical impacts included the consequence of the cancer experience on everyday tasks, activities, routines, and lifestyle. Many participants reported inability, uneasiness, or difficulty performing daily activities they used to be able to do, such as being unable to travel, reading, playing with Legos, motorcycling, playing musical instruments, activities with their children, and others. Several men spoke of the impacts of cancer on sports and physical activities, some of which were long-term, with engagement in physical activities generally not returning to prediagnosis levels. Some men discussed the impacts of cancer on routine home maintenance tasks (e.g., household chores, cleaning up, changing the car’s tires, mowing the lawn, or renovating the house). Treatments often involved occupational impacts, resulting in an interruption of work and school ranging from a few days to a few years in the form of sick leave, early retirement, disability period, or loss of employment. Some men found it challenging to return to work after completing treatments. This was due, for example, to having been replaced or to the persistence of residual symptoms. Reduced availability and limits on the tasks men could perform at work pushed some of them to eventually stop working.

Cancer thus was challenging to the financial situation of many men. For some, loss of income and increased spending (e.g., hospital parking, supportive care) was a significant drain on their finances and savings, which was not entirely replaced by the financial aid available. Difficulties men experienced with finances mainly were related to their capacity to provide for themselves and their families, including difficulties in covering rent, making car payments, and paying their mortgages.

#### Spiritual Impacts

Spiritual impacts included effects of the cancer experience on men’s religion and spiritual beliefs and practices. Only a few men reported that their spiritual life was impacted. One of them explained that his cancer experience, particularly in terms of interactions with other patients at the hospital who would later pass away, confirmed his atheism. Another man explained that he now believed in the possibility of God existing, although he was still not practicing a particular religion.

### Strategies to Cope With the Experience of Cancer

Strategies were defined as actions, attitudes, or beliefs men and others put into place to help cope with, manage, or improve men’s experience with cancer. They were also categorized into physical, psychological, interpersonal, informational, practical, and spiritual domains. These categories were defined and refined from data. Table 3 provides strategies identified in each domain along with illustrative examples and quotes from participants.

#### Physical Strategies

Physical strategies were actions involving physical processes or mechanisms, as well as procedures, interventions, or treatments on one’s body or physical health. They include formal and informal help with physical intervention goals, such as medical support from health professionals. All men received enormous medical support from hospital services and professionals. Men discussed support from professionals and services such as pharmacists, dentists, nurses, physicians, nutritionists, physical therapists, and occupational therapists. They discussed medication, medical devices, and procedures such as crutches, urinary catheters, orthoses, ostomies, and prostheses. Several men benefited from other services such as massage therapy, acupuncture, various fitness programs, osteopathy, physical rehabilitation, and smoking cessation help from a community organization. Also, men mentioned ways they were engaged in, and making decisions regarding their medical cancer care process, such as refusing pain relievers, and autonomy for their treatment. Some men indicated making decisions concerning symptoms, such as self-assessment, self-diagnosis (for a participant who was a health professional), and consulting health professionals when new symptoms appeared. They discussed acting on their medical preferences, such as requiring face-to-face visits with their physician, asking to change physician when not a good fit, or generally sharing their preferences with medical staff.

Men discussed engaging in various healthy habits and behaviors aimed at managing physical impacts, and ways they took care of themselves and took their health seriously. Although some tried to get back into shape or improve their body, others took time to heal or rest. Some men paid attention to their limits by planning according to their daily strength, taking it easy, or preserving their energy for certain activities. For some men, having a healthy lifestyle was important, as they felt it improved their chances of recovering from cancer. Efforts related to nutrition were varied, including restrictions, low-carb diets, supplements, fasting, increased fruits and vegetables, and other changes. Health professionals encouraged some of these changes, while others were adopted as a result of word-of-mouth or internet research. A few men indicated stopping drinking alcohol and smoking tobacco. One man discussed oxygenation exercises he engaged in to help with respiration. Other changes were made by an immunocompromised man, such as having his own bathroom and making sure his daughter disinfected her hands to eliminate germs.

#### Psychological Strategies

Psychological strategies involved engaging in psychological processes or mechanisms, usually to minimize, manage, or reduce unpleasant emotions. They include formal and informal help with psychological and emotional intervention goals. Psychological support sought or received by most men came in large part from psychosocial professionals and services. Although psychologists were consulted by around half of the men, social workers, support groups, peers, and art therapy were also mentioned. In a few cases, men indicated receiving support from a psychiatrist or receiving medication (i.e., antidepressants and antipsychotics). Although men gave very few specifics concerning social workers and art therapy, they indicated partaking in support groups and support dyads with other cancer patients. Professionals facilitated formal support groups and patient pairings and could include spouses. Informal groups consisted of Facebook support groups that one man looked for, joined, and participated in. For some men, nurses and community organizations also provided them with psychosocial support. Many men benefited from support from people who would comfort, reassure, and encourage them during this difficult experience. Friends, family, and health care professionals would comfort men by telling them not to worry or normalizing symptoms experienced. In one case, a man was comforted by a friend normalizing his sexual difficulties. Men would receive encouragement, especially from family members, telling them they can go through this experience. Men also reported that the encouragement and admiration they received from others were instrumental in their recovery and induced positive feelings.

Several men discussed psychological processes they engaged in to cope, including adopting a particular perspective and mind-set, being future-oriented, or talking about cancer in the past while still in treatment. While only one man indicated being pessimistic about his situation, almost all others engaged in a positive, optimistic, or hopeful outlook. Men overwhelmingly preferred to be positive and reflect positively on their experience—and thought that doing otherwise was counterproductive. Acceptance was a common theme. Many men indicated accepting their lack of control over their situation, acknowledging that it occurred due to outside influences. Defense mechanisms were prevalent. Although some men reported a process of acceptance and normalization of their situation and experience, others discussed the difficulty of accepting some aspects of it, such as physical impacts or pain.

Men often indicated using avoidance to help them manage their experience. Some men would avoid discussing their diagnosis or thinking about what was happening to them. A few men reported practicing emotional restraint, not allowing themselves to feel emotions or allowing themselves a short time to “feel depressed.” They discussed avoiding engaging in some negative thoughts (e.g., thinking about their illness, or their financial situation) and avoiding seeking help (e.g., sex therapist). In these cases, men sometimes used what could be considered self-persuasion. Indeed, some men spoke of times when they actively told themselves unfounded positive future events and news to make themselves feel better. For example, some men said they convinced themselves that they were recovering or told themselves it was not possible for them to die. Some actively persuaded themselves that their experience was temporary, that everything was going well for them, or that they would be able to go back to their normal life at some point. In the case of one man experiencing sexual impacts, convincing himself that sexuality was not the most important thing in life was also discussed.

A few men described using humor as a way to cope. They discussed using self-deprecation comments and jokes about the situation. Men engaged in different thoughts during their experience of cancer. They indicated reflecting on several things, placing their priorities, and assessing their situation. Focusing on their recovery would also help some men, though others would turn their attention to their loved ones. In some cases, men indicated that their way of coping with psychological impacts and stressors was to put their trust and confidence in health care professionals. A few men demonstrated resilience in their experience by using their own resources to help them cope psychologically.

Men engaged in different self-regulation behaviors to help them cope with or manage their experience of cancer. Many men indicated being in a general state of action; they discussed being task-oriented, solution-focused, and wanting to know what to do “rather than crying and feeling sorry for themselves.” Men used different ways of expressing being proactive and “fighting against,” or more often preferred, “resisting” their illness. During their experience with cancer, these men generally sought to take or be in control. Some men reported respecting their limits by listening to themselves and going at their own pace. A few men discussed crying, particularly during the announcement of the diagnosis. Interestingly, many men who discussed crying used particular turns of phrase that minimized this behavior (e.g., “almost cried,” “cried internally,” “tears came,” “having cried at times”). Men discussed relaxing, meditating, and putting effort into removing stress from their lives. Keeping light in a room to keep morale up and doing something good and pleasurable for themselves (“treating yourself”) were mentioned.

#### Interpersonal Strategies

Interpersonal strategies were interpersonal actions by men or others involving social associations, connections, or affiliations between two or more people motivated by human needs such as belonging, closeness, intimacy, or trust. Men discussed many ways in which being connected with others was beneficial to their experience with cancer. First, men presented many ways in which being in the presence of others was sought or appreciated. A majority of men described people as “being there for them,” but also valued others accompanying them to their medical appointments, information sessions, and treatments. Friends and family would visit men, sometimes at the hospital. Some men made substantial efforts to see loved ones by returning to their families in their hometowns or visiting their children overseas. A few men indicated that being with the same patients in the waiting rooms during their recurring appointments was comforting. For some men, however, the presence of others was avoided. To deal with their experience, men sometimes preferred to be alone or take a step back from others.

Almost all men mentioned social connections with others. One way to be connected with others was to share their diagnosis and related information, updates, and prognostics with people they feel close to. Some men indicated they shared the news quickly with their family and friends, while others waited a bit, often to prepare themselves. Men who quickly shared news sometimes discussed how they wanted to be the ones announcing their diagnosis rather than having it disclosed by someone else. Men discussed how they disclosed some of the impacts the cancer experience had on them, particularly in the context of impotence when dating. On the contrary, men also sought to limit or restrict sharing with others; they were numerous to indicate that they chose carefully whom they wanted to tell about their cancer. Men often disclosed to close friends and family members, though the information was also withheld from family members they deemed too fragile (e.g., grandparents). Some men made lists of people to tell or not, lied to some people by saying they were doing well, and avoided questions regarding their situation. In some cases, men reported not getting invested in intimate relationships to protect the other person in the event of adverse events related to their prognostics. A few men indicated not wanting to talk or exchange with others, often regarding their situation. Others indicated choosing the people to exchange with wisely; negative people, usually other patients, were strongly avoided by men. In general, the theme of social belongingness was somewhat prevalent in the discourse of men. Having a good social network and fostering existing relationships were strategies that were deemed helpful. In addition, a good proportion of men described social behaviors of understanding, affirmation, and demonstrations of caring akin to intimacy. Men perceived others as caring by giving them attention, encouragement, and consideration.

During their experience with cancer, men discussed managing other people and handling their relationships in different ways. Men indicated benefiting from helping others. While a few men discussed helping family members with various daily tasks (e.g., administrative tasks, homework), many regarded helping new cancer patients and fellow men as rewarding. However, the experience of cancer also had numerous impacts on people around men. Men found themselves in difficult positions when they had to manage others’ emotions. They reported paying close attention to what they communicated to their family and friends so as to not worry them. When they had children who were told about their condition, men reassured them and prepared them for their upcoming treatments. Anxious spouses were comforted. For some men, telling other people you are doing well when you are not, responding with optimism when people show empathy, and making jokes when people ask about their cancer were all strategies they used to manage others. In these cases, however, these strategies were not used to deal with the emotions of others but rather to avoid talking about cancer, not being perceived as a burden, and putting on a brave face. In other cases, men indicated putting their limits with people. For example, one man explained that making it clear to people that they are not always available was essential.

#### Informational Strategies

Informational strategies were actions and processes of receiving and seeking knowledge, gaining skills, and developing abilities during the experience of cancer. Managing information was one of the most prominent strategies men used. Most men took an active role in gathering information from various sources by asking questions, seeking advice, and look for additional information. Health care professionals, in particular, were solicited for information regarding cancer and available support services. Pivot nurses (i.e., specialized oncology nurses who provide patients and their families with continuing and consistent supportive care throughout the care continuum) were helpful in answering questions quickly without making an appointment with their physician. For some men, preparing themselves by gathering information through research, preparing questions, and making lists of possible treatments before meeting their physician, was helpful. One man indicated preparing himself by gathering information before returning to his family and announcing his diagnosis to them. Men mentioned getting their information from other sources, with the internet being an important source for some. Other sources include magazines and books (e.g., running magazines), scientific sources (e.g., medical scientific watches, scientific articles), and universities (libraries, webinars). Some men discussed seeking informational support from other cancer patients through cancer discussion groups, particularly on Facebook. Experimental and alternative forms of treatments were shared there.

In some ways, men have described how restricting cancer information was used as a strategy. In this case, men have first discussed not looking up information online as not all information available online was truthful or helpful. Some explained that this was encouraged by their physician. In fact, some men indicated feeling that health care professionals sometimes withheld some information themselves. In other cases, men restricted information themselves because they did not want to receive it. Some indicated not asking questions regarding diagnosis and treatments, or even avoiding information such as prognostics.

Throughout this experience, men have sought to learn, expanding their knowledge of fields and subjects related to their condition. Watching YouTube videos on different educational fields and scientific subjects, looking up scientific topics, and taking online university courses related to health were all strategies employed by men to learn. They found it essential to share this information and advice with friends, family, men, and other cancer patients.

#### Practical Strategies

Practical strategies were tasks, activities, and situations of the usual routine in which men or others engaged in to help cope with and manage the experience of cancer or make one feel better, including support and actions related to work, household responsibilities, sports, and hobbies. Men have discussed engaging in numerous activities throughout their experience with cancer. They reported a wide range of physical activities, sports, and exercises, including walking, running, soccer, yoga, skiing, golfing, stretching, and strength training. For some, it was an opportunity to begin and try new activities, and make efforts to do more exercise, most often with an objective related to physical health or recovery. During their experience, men have engaged in leisure activities and hobbies, including games (e.g., videogames, Legos, bowling), visual art (e.g., photography, sculpture, painting, drawing), music (e.g., listening to music, playing instruments), reading, writing, watching TV, and traveling. Men also privileged outdoor activities such as motorcycling, camping, fishing, gardening, and snowmobiling. Some of these activities were shared with loved ones, such as their spouse or children. Continuing activities and tasks they enjoyed and could do was very helpful to some men, partly to keep themselves busy and avoid negative thoughts. For some, then, being active around the house was enjoyed. Shoveling snow and house renovations were some of the chores that helped men feel good, although not always recommended by their physicians. Several men talked about wood chopping, construction work, and repairing as tasks they enjoyed and provided them with pleasure. A few men have discussed their involvement in volunteering activities as something that helped them. While some discussed volunteering for different causes unrelated to their cancer, others have found a use for their experience by getting involved in cancer support organizations.

Financial support was important for men who had to stop working during their experience. Men discussed receiving financial relief from insurance and government aid such as social assistance, disability pensions, unemployment insurance, and sickness unemployment benefits. Some men found that the amounts received and the eligibility period were insufficient to help people with cancer, as the cancer continuum could last years. Other strategies included, for example, selling their house, renting more economical housing, budgeting, and engaging in bartering services with others to save money. Friends, family, and coworkers have also helped men financially. In one case, a man facing costly experimental treatment fees was supported by a GoFundMe funded by family, friends, and co-workers. He explained that the annual funding event for charities organized at his workplace was used to pay for his treatments this year. Finally, some men relied on their savings to fund their expenses related to treatment and work interruption.

Since numerous men interviewed still had an active professional life, occupational adjustments were discussed by many. Some indicated continuing with their work activities or quickly returning, but for others, the ability to take some time off work greatly helped them deal with the cancer experience. Support from employers was necessary; being understanding and accommodating was appreciated by men going through cancer. Other examples of strategies that helped men included taking classes online, working from home, and having a low-impact office job.

Men received help with household chores and tasks. They reported support for preparing meals, doing laundry, mowing the lawn, doing the groceries, taking care of the children, and cleaning, mostly from their parents and their spouse, but also from friends. Having friends and family drive them to the hospital was appreciated, as some had to take a taxi to get to the hospital.

#### Spiritual Strategies

Spiritual strategies were religious and spiritual actions, views, and perspectives used by men concerning their experience of cancer. Although few men mentioned religion as a strategy to cope and manage their experience, it was prominent strategy for those who did. Such a spiritual mind-set helped some men cope with difficult emotions. Specifically, these men described various behaviors and activities associated with organized religions, such as going to a place of worship, reading sacred texts, and praying. Some of these men have indicated that religious figures (i.e., God, Jesus) decided their fate and that their recovery depended on them. Religious figures were also responsible for helping them in their experience of cancer. One man described great trust in his ability to heal. This man mentioned receiving spiritual support from a religious nurse at the hospital he thought may have been sent by God. He recalled the nurse telling him to pray and trust the Lord, which he appreciated.

## Discussion

This study examined the experience of men diagnosed with cancer, focusing on the impacts of the illness and its treatment, and on men’s strategies to cope with the impacts. Findings underscore the diversity of impacts and strategies used by men, support the need for holistic and personalized support services, and highlight the valuable resources men hold that can be drawn upon to support them throughout the cancer care continuum.

Numerous impacts of cancer, including symptoms and consequences of treatment, were discussed by men. Cancer diagnosis and treatment affected men in all domains of their lives, although they reported fewer spiritual and informational impacts. This is consistent with other studies indicating that physical, psychological, and interpersonal impacts were prevalent in patients (Bryant-Lukosius et al., 2010; Carter et al., 2011; Fitch, 2012; Fitch et al., 2021). However, current care is often centered on physical needs, and access to supportive care services in oncology is limited and depends almost exclusively on services offered by the hospital and the community organizations present (Cardoso et al., 2013; Carter et al., 2011; Kirkman et al., 2017; Montiel et al., 2023; Zafar et al., 2022). These results support the need for a holistic approach to cancer, in which needs are seen as being varied and depending on personal characteristics and preferences, cancer diagnosis, treatments, and prognosis (Montiel et al., 2023). A focus on achieving a better understanding of personal needs could be appropriate for physicians, nurses, physiotherapists, kinesiologists, psychologists, sex therapists, social workers, and others supporting this population.

The current results pertaining to strategies to cope with cancer impacts also highlight an active and resourceful stance adopted by male cancer patients. Indeed, many men discussed selected positive impacts of cancer on their lives, such as learning new skills, strengthened relationships, changes in attitudes, and more positive outlooks. Such results indicate that in the face of adversity, men often demonstrate resilience and mobilize their strengths to help them get through the experience (Wenger & Oliffe, 2014). Men discussed a wide range of strategies used to help them cope with cancer. In addition to the importance of medical support, psychological support was often sought and appreciated, which is not made clear by studies reporting low formal help-seeking in men with psychological distress in the general population (Barney et al., 2006; Biddle et al., 2006; McKenzie et al., 2018; Montiel et al., 2022; Nam et al., 2010). It is possible that some factors related to the experience of cancer and services explain these findings, such as referrals provided by physicians (Montiel et al., 2023). Coherent with previous studies (Mosher et al., 2015; Spendelow et al., 2018), men in our study indicated using many coping strategies that are deemed ineffective in the literature, such as denial and avoidance. However, they also discussed more adaptive coping strategies, including positive reframing, acceptance, and humor. These results suggest that research and practice may gain from adopting a more positive, empowering, and strength-based approach to supporting men’s coping with the impacts of cancer diagnosis and treatment.

Men discussed many hobbies and tasks they enjoyed doing such as games, fishing, reading, and house renovations. Engaging in usual activities has been included in several questionnaires concerning coping with cancer and is considered a way to redirect attention, thoughts, or behavior away from negative thoughts and feelings (Cwik et al., 2021; Moorey et al., 2003). Several studies have identified that engaging in these kinds of activities is a way for men to carry on as normal and not let cancer interfere with their lives (Gray et al., 2000; Wenger & Oliffe, 2014), or to take control (Spendelow & Seidler, 2020). Cutting trees down, splitting wood, flying an airplane, running errands, riding a motorcycle, and gardening were some of the activities discussed by patients with late-stage cancer by Cheville et al. (2012) as usual physical activities they privileged over formal exercise programs. Cancer patients have also discussed activities such as walking, gardening, and hobbies as helpful in alleviating fatigue (Spichiger et al., 2012).

Men described many supportive actions from others, such as bringing homecooked meals, doing household chores, and mowing the lawn. Men appreciated being with others and keeping in contact with them, highlighting the importance of interpersonal relationships. These results suggest that, although men are often reported to be self-reliant and refuse formal help (Möller-Leimkühler, 2002), they appreciate particular forms of support from others. They carefully select sources of interpersonal support by limiting who and how many people may know about their condition (Gray et al., 2000). In this study, simply being in the presence of loved ones, staying in contact, and keeping their relationships “normal” echoed themes from previous studies (Gray et al., 2000). In fact, maintaining a status quo and using existing resources, in part by resisting changes in social roles, was highlighted by Spendelow and Seidler (2020) and Wenger and Oliffe (2014) as one of the main coping strategies in men. Further research is needed to establish in which context, when, and why men accept to receive help from others. Men engaged in a wide range of activities to cope with impacts from interconnected domains of their lives. Impacts in one life domain may call for strategies from a variety of domains, and conversely, strategies from a single domain can be deployed to better manage the impacts of various domains. It also highlights the difficulty in assessing coping strategies in patients and providing standardized recommendations.

The various impacts of cancer and the resulting strategies used by men suggest that these impacts are multifaceted and contain elements that may be beyond the scope of professional (i.e., nonexperiential) knowledge. Men diagnosed and treated for cancer appear to possess and cultivate strengths, knowledge, and competencies that are mobilized during the experience of cancer but that are seldom recognized (Jackson et al., 2023). A holistic perspective necessitates a coordinated and integrated approach to user involvement in cancer services to provide personalized and individualized care. This supports the necessity of adopting approaches to care in which patients occupy a more central role, such as a *patient–partnership* approach that considers patients and their family members as full members of the health care team with complementary perspectives to the health care professionals’ (Karazivan et al., 2015; Pomey et al., 2015). An effective patient–partnership in health care involves collaborating with the user, their family, and health care professionals in an interactive and learning-focused process. This helps to promote the user’s self-determination, free and informed decision-making, and achievement of optimal health outcomes. It allows health care professionals to view the patient as a complex individual whose needs go beyond medical diagnosis and treatment. Men in this study expressed the importance of being included in their health care pathway and their own recovery (Montiel et al., 2023), highlighting the need for their experience and knowledge to be harnessed to support and empower them during diagnosis and treatment for cancer. Future efforts should continue to integrate such partnership approaches to cancer care and aim to provide personalized care which addresses the multitude and diversity of men’s needs.

## Limitations

Categorizing impacts and strategies through interconnected life domains yielded some disadvantages regarding theoretical conceptualization and classification. For example, interpersonal strategies excluded the social support attributes commonly found in the literature, such as emotional, informative, appraisal, and instrumental support (Langford et al., 1997). In our study, psychological, informative, or practical support from others were not counted as interpersonal strategies and were classified in their respective domains. This decision was made to avoid an unbalanced and theoretically ambiguous conceptualization of interpersonal strategies including all types of support and actions from others. Other examples include physical activity being classified as a practical strategy, in line with the definition used centered on the activity, while it could have been part of physical strategies of lifestyle behavior changes. Finally, the spiritual domain was elaborated from previous conceptualizations of spirituality as a “personal, subjective side of religious experience” (Hill & Pargament, 2003) and a general focus on religious strategies in the literature (Pearce et al., 2012). Philosophical elements concerning life meaning and appreciation were therefore classified into the psychological domain. However, we recognize that it represents a very Western-centered classification of many impacts and strategies that have been discussed by men.

Our study used a convenience sample composed of *men* who volunteered to participate in response to advertisements. While our inclusion criterion was not restricted to individuals assigned male at birth, our participants were all cisgender men. This may be explained by the low prevalence of transgender men, social stigma, and our potentially gender-normative advertisement (i.e., not specifying “self-identifying” men, images of men with conventional masculine undertones). As such, the male gender experience captured by this study is limited. The experience of transgender men with diverse cancer diagnoses merits further research (Squires et al., 2022). It is also possible that participants represented a unique profile, possessed more resources, and demonstrated a larger capacity to self-reflect, open themselves to others, and share. However, a strength of the study resided in the use of both interviews and focus groups, providing us access to participants with different profiles and preferences in terms of social and group-based activities. Although our study provides an in-depth understanding of the experience of Québec men with cancer, findings are likely to be contextually bound. Our study provides emerging insights rather than an overarching explanation of the experience of all men with cancer.

## Conclusion

Our understanding of men’s experience of cancer and the resulting needs is limited as evidenced by low use of supportive care services. This study provides new insights into the diverse impacts men face across various domains of their lives and the coping strategies they employ from a range of other domains. The findings highlight the importance of considering impacts beyond the physical realm and shed light on the diverse and multiple aspects of men’s responses to the challenges posed by cancer. Understanding and leveraging these strategies can inform the development of personalized supportive care interventions that resonate with men’s strengths and preferences, ultimately enhancing their coping abilities and overall quality of life. The contribution of the current research resides in its comprehensive, strengths-based and holistic portrayal of men’s experience with cancer diagnosis and treatment, which can inform supportive care resources and services.

From the findings of this study, clinicians can gain insight into the complex and often unmet supportive care needs of men diagnosed and treated for cancer. Men reported diverse impacts of cancer on their physical, psychological, and interpersonal domains, such as negative affect and relationship challenges resulting from the experience. In addition, the study revealed that men engage in various strategies to cope, including psychological and interpersonal support, which are not typically associated with men. Addressing complex individual supportive care needs can be challenging for health care professionals. Therefore, adopting a comprehensive and holistic approach to personalize support for men in coping with the impacts of cancer diagnosis and treatment is essential. Personalized medical and supportive care services can benefit from working in partnership with patients, and recognizing and identifying men’s unique strengths and available resources.

## Supplemental Material

sj-docx-1-jmh-10.1177_15579883231215153 – Supplemental material for “In My Mind, It Was Just Temporary”: A Qualitative Study of the Impacts of Cancer on Men and Their Strategies to Cope Click here for additional data file. Supplemental material, sj-docx-1-jmh-10.1177_15579883231215153 for “In My Mind, It Was Just Temporary”: A Qualitative Study of the Impacts of Cancer on Men and Their Strategies to Cope by Corentin Montiel, Nathalie Bedrossian, André Myre, Asher Kramer, Alexia Piché, Meghan H. Mcdonough, Catherine M. Sabiston, Anika Petrella, Lise Gauvin and Isabelle Doré in American Journal of Men's Health
